# MxlPy—Python package for mechanistic learning and hybrid modelling in life science

**DOI:** 10.1093/bioadv/vbaf294

**Published:** 2025-11-18

**Authors:** Marvin van Aalst, Tim Nies, Tobias Pfennig, Anna Matuszyńska

**Affiliations:** Department of Biology, Computational Life Science, RWTH Aachen University, Aachen 52074, Germany; Department of Biology, Computational Life Science, RWTH Aachen University, Aachen 52074, Germany; Department of Biology, Computational Life Science, RWTH Aachen University, Aachen 52074, Germany; Cluster of Excellence on Plant Sciences (CEPLAS), Düsseldorf 40225, Germany; Department of Biology, Computational Life Science, RWTH Aachen University, Aachen 52074, Germany; Cluster of Excellence on Plant Sciences (CEPLAS), Düsseldorf 40225, Germany; Center for Computational Life Sciences, Aachen 52074, Germany

## Abstract

**Summary:**

Recent advances in artificial intelligence have accelerated the adoption of machine learning (ML) in biology, enabling powerful predictive models across diverse applications. However, in scientific research, the need for interpretability and mechanistic insight remains crucial. To address this, we introduce MxlPy, a Python package that combines mechanistic modelling with ML to deliver explainable, data-informed solutions. MxlPy facilitates mechanistic learning, an emerging approach that integrates the transparency of mathematical models with the flexibility of data-driven methods. By streamlining tasks such as data integration, model formulation, output analysis, and surrogate modelling, MxlPy enhances the modelling experience without sacrificing interpretability. Designed for both computational biologists and interdisciplinary researchers, it supports the development of accurate, efficient, and explainable models, making it a valuable tool for advancing bioinformatics, systems biology, and biomedical research.

**Availability and implementation:**

MxlPy source code is freely available at https://github.com/Computational-Biology-Aachen/MxlPy. The full documentation with features and examples can be found here https://computational-biology-aachen.github.io/MxlPy.

## 1 Introduction

Mathematical modelling has evolved into a cornerstone of modern biological research, offering a rigorous, quantitative framework to explore the complexity of living systems. However, the ongoing artificial intelligence revolution is reshaping the computational biology landscape, where small-scale mechanistic models are increasingly being displaced by data-driven predictive approaches. In line with recent perspectives (Baker *et al.* 2018), we believe that life sciences should embrace the complementary strengths of both mechanistic and machine learning (ML) methods. Motivated by this vision, we decided to develop software that supports hybrid modelling approaches, in particular, mechanistic learning, which combines interpretability with predictive power.

### 1.1 What is mechanistic learning?

**Figure 1.**
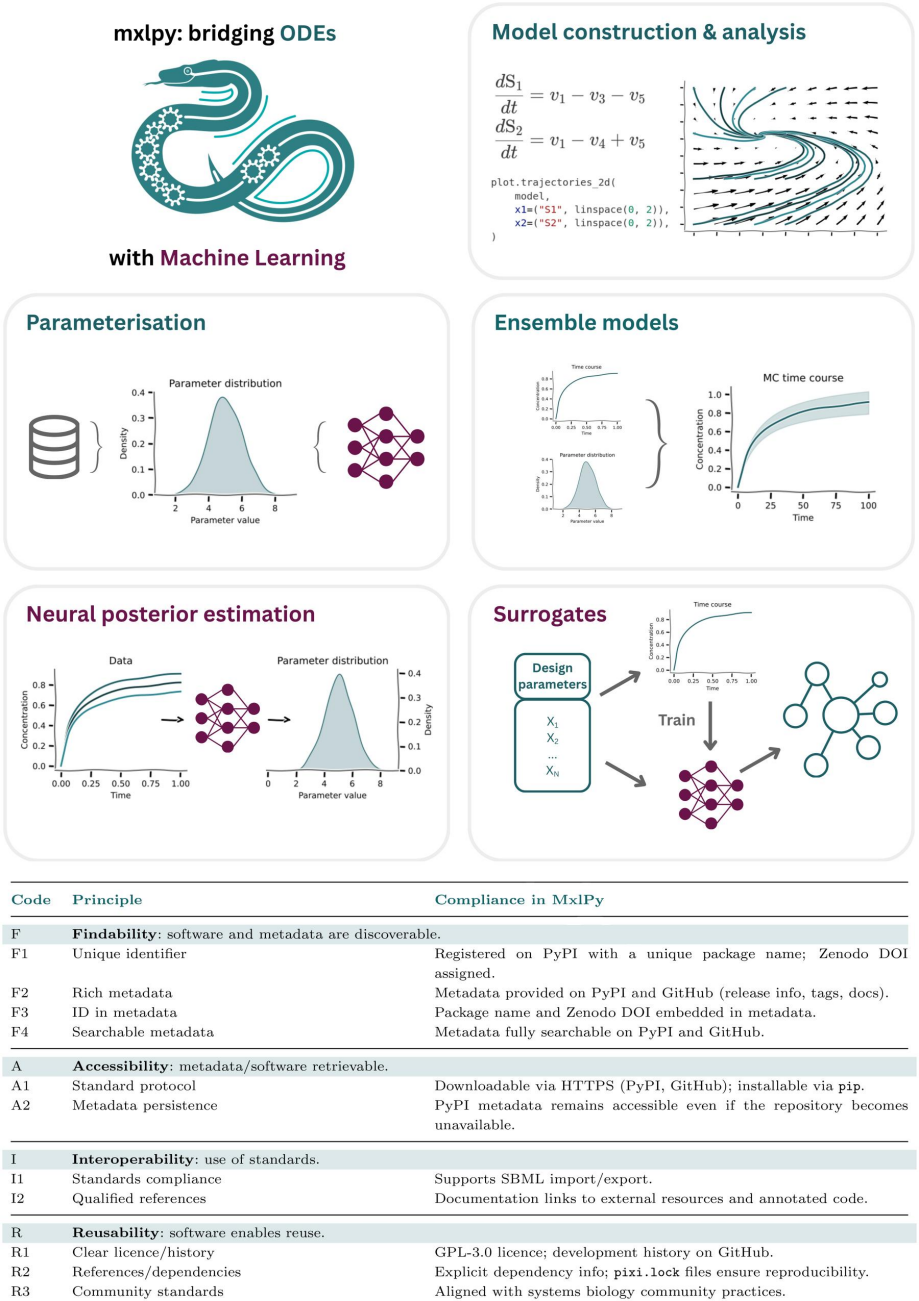
Key features of MxlPy enabling integrative kinetic modelling and machine learning and their alignment with FAIR4RS principles. MxlPy facilitates mechanistic learning by combining traditional kinetic modelling with modern machine learning techniques. The platform supports direct parameter extraction from databases such as BRENDA, ensemble modelling for uncertainty quantification, and integration of surrogate models and neural posterior estimation methods. Designed for both experts and non-specialists, MxlPy streamlines parameter management, automates Monte Carlo simulations, and enables the hybridization of data-driven and mechanistic approaches for systems biology applications.

Mechanistic learning (MxL) is an emerging approach that combines two fundamentally distinct modelling paradigms: mechanistic modelling and ML (Metzcar *et al.* 2024). Rather than treating data-driven and knowledge-driven approaches as separate tools, mechanistic learning combines the strengths of both approaches: using ML to infer unknown relationships, calibrate models, or augment incomplete mechanisms, while grounding model behaviour in the structure and interpretability of mechanistic equations. This idea shares conceptual ground with related fields: scientific ML (Thiyagalingam *et al.* 2022), used predominantly in physics and engineering, emphasizes embedding physical laws into ML models; or hybrid modelling more loosely refers to any combination of mechanistic and data-driven approaches (Schweidtmann *et al.* 2024). What distinguishes mechanistic learning, especially in the life sciences, is the deliberate integration of ML into mechanistic modelling pipelines to complement and inform the model structure itself. In their review, Metzcar *et al.* distinguish four types of mechanistic learning (Metzcar *et al.* 2024): sequential, parallel, extrinsic, and intrinsic. In the sequential type, the output of either a kinetic model or an ML approach is fed into the other as input. For instance, researchers developed neural networks (NNs) to estimate parameter values for enzyme-catalysed reactions, which could be used in a mechanistic model (see Kroll *et al.* 2021). Parallel mechanistic learning treats ML and mechanistic modelling as equivalent. Here, NN can serve as surrogate models that can replicate input-output relationships similar to mechanistic models without a deeper understanding of the underlying biology. Using surrogates allows researchers to make faster predictions by evading computationally costly differential equation integration (Gu and Ng 2023). The extrinsic approach employs mechanistic modelling for understanding post hoc what NNs have learned. Finally, intrinsic mechanistic learning uses domain knowledge in the development of statistical models [e.g. physics-informed (PINN) (Cuomo *et al.* 2022) or biology-informed neural networks (BINN) (Lagergren *et al.* 2020)]. Overall, mechanistic learning can be regarded as an approach to constructing digital twins or multiscale models (Gruber et al. 2024, Lorenzo et al. 2024, Noordijk *et al.* 2024, Lan *et al.* 2025). Despite its promise, mechanistic learning remains largely inaccessible to beginners and researchers without a strong computational background. Currently, few software tools support the seamless integration of NNs into mechanistic models. In most cases, such integration is implemented *ad hoc* within individual projects, without reusable, user-friendly infrastructure, e.g. (Renardy *et al.* 2018, Madani *et al.* 2019, Führer *et al.* 2024) (with some notable exceptions, discussed in the Table 1, available as supplementary data at *Bioinformatics Advances* online) (Hoops *et al.* 2006, Blinov *et al.* 2017, Choi *et al.* 2018, Leweke and von Lieres 2018, Merkelbach *et al.* 2022, Loman *et al.* 2023). To bridge this gap, we developed MxlPy. It introduces both sequential and parallel mechanistic learning workflows, specifically designed to address the common challenge of missing or uncertain information in mechanistic models. With a clean, Pythonic interface, MxlPy empowers not only experts but also students and experimentalists to perform neural posterior estimation (NPE), assess parameter uncertainty using Monte Carlo (MC) methods, integrate surrogate models, or retrieve biologically relevant parameter distributions from open-access databases (see Fig. 1). By lowering technical barriers and embedding powerful inference tools, MxlPy makes mechanistic learning truly approachable for the broader life science community.

## 2 Methods and results

We present the core functionalities of MxlPy, a Python-based framework designed to integrate ML techniques with mechanistic modelling in the life sciences. MxlPy extends the capabilities of its predecessor, modelbase (van Aalst *et al.* 2021), a toolkit tailored for building and simulating mechanistic models based on ordinary differential equations (ODEs). The current implementation of MxlPy provides five primary modules: mechanistic model construction and analysis, ensemble modelling (parameter sampling), surrogate model integration, parameter assessment from databases, and NPE (Fig. 1). In line with FAIR4RS principles (Barker *et al.* 2022), MxlPy is openly available as a community-driven project: https://github.com/Computational-Biology-Aachen/MxlPy. A comprehensive and regularly updated technical guide, including reproducible examples, is available via the official MxlPy documentation portal: computational-biology-aachen.github.io/MxlPy.

### 2.1 Embedding FAIR and CURE principles into the modelling cycle

Ensuring that computational models adhere to the FAIR: Findable, Accessible, Interoperable, Reusable (Barker *et al.* 2022) and CURE: Credible, Understandable, Reproducible, and Extensible (Sauro *et al.* 2025) principles is not something that should be addressed retroactively at the end of a project, but rather embedded throughout the entire modelling cycle. Critical aspects such as metadata, unit consistency, transparent code, and explicit documentation need to be integrated from the first steps of model construction through to analysis and dissemination. MxlPy has been designed with this philosophy in mind: its architecture actively supports FAIR and CURE compliance of the developed models by enforcing unit checks, generating metadata, and maintaining code transparency within the workflow itself (table within Fig. 1). This approach is particularly important in life-science collaborations, where experimental and computational partners must exchange models and data seamlessly, and where reproducibility and clarity are essential for cross-disciplinary trust. In this way, reproducibility and community reusability become natural by-products of the modelling process, rather than an additional burden once the research is complete (Sauro *et al.* 2025).

### 2.2 Modularized construction and analysis of kinetic models

Kinetic models in MxlPy are built programmatically using plain Python functions. The model objects provide full introspection and other meta-programming capabilities. These functionalities allow models to be built interactively and reuse parts of or the whole model in different computational projects, enabling modularization via composition and inheritance. The key building blocks of kinetic mechanistic models [Equation (1)] are parameters *k*, variables *S*, and reactions specified by rate laws v(t,S,k) (*t* is simulation time), and stoichiometries *N* (stoichiometric matrix):


(1) $$\frac{dS}{dt}=Nv(t,S,k).$$

These building blocks and the simulation time can be used to derive additional stoichiometries, parameters, and variables. The type (static or dynamic) of derived quantities is determined automatically using the programmatic concept of lazy evaluation. All these model components can be annotated with units, and their consistency can be checked to enable early detection of common bugs, mismatched concentration and flux units, inconsistent time scales, or missing compartment scaling. Easy-to-use methods guide users through the specification of all parts of a computational model, allowing a gentle learning experience. MxlPy allows simulation of dynamic variables both under standard conditions and under protocol-driven scenarios, where parameters change during the simulation according to predefined switching rules. The underlying ODEs of models are integrated using solvers implemented in the Python packages SciPy (e.g. LSODA) (Virtanen *et al.* 2020) or Assimulo (CVode) (Andersson *et al.* 2015). Users can fully customize all solver parameters, thus handling dynamically challenging integrations (e.g. stiff problems). Several advanced functionalities are implemented in MxlPy, like steady-state analysis of biological systems through metabolic control analysis (Wildermuth 2000). Additionally, a LaTeX export feature is provided to facilitate the streamlined reporting of model structure by program introspection, providing ready-to-paste systems of equations for scientific publication, theses and other technical documents. A novelty in MxlPy is the automatic transformation of dynamic models into symbolic form using the SymPy library (Meurer *et al.* 2017). This symbolic representation enables integration with advanced mathematical frameworks for structural analysis of biochemical networks, including stoichiometric analysis, linear stability analysis, and structural identifiability. Structural identifiability analysis determines whether the parameters of a model can be uniquely estimated from noise-free, continuous observations, given the structure of the differential equations alone (Wieland *et al.* 2021). Structurally unidentifiable parameters are inherently non-estimable regardless of the data quality or quantity (Cobelli and DiStefano 1980, Wieland *et al.* 2021). Detecting such parameters is essential in systems biology, particularly for interdisciplinary projects where precise parameter estimation underpins experimental validation and mechanistic insight. MxlPy integrates StrikePy, currently the only fully Python-based tool for structural identifiability analysis (Rey Barreiro and Villaverde 2023). StrikePy uses symbolic computation to evaluate identifiability in small to moderately sized ODE models, thus complementing MxlPy’s symbolic infrastructure.

### 2.3 Parametrization

MxlPy provides user-friendly methods for extracting and integrating values of kinetic parameters from the BRENDA enzyme database (Chang *et al.* 2021) into models. A copy of an enzyme’s entry or a set of entries must be downloaded from the database as a JSON file. Functionalities in the MxlPy’s parameterise module help extract the desired information from the downloaded database entries and provide the user with a data frame that facilitates further analysis.

### 2.4 Ensemble modelling and Monte Carlo sampling

MxlPy provides a dedicated framework for ensemble simulation using MC sampling through its mc and distributions modules. Users define the uncertainty in each model parameter by specifying appropriate statistical distributions (among others, uniform, normal, log-normal), e.g.


(2) $$\begin{array}{c} k_{0}∼N(\mu ,\sigma^{2}) \end{array}$$

These distributions can be derived from domain expertise, experimental measurement ranges, or extracted directly from biological databases [e.g. BRENDA (Chang *et al.* 2021)] when multiple empirical values are available for a given parameter across organisms or conditions. Once the parameter distributions are specified, MxlPy performs repeated sampling to generate a user-defined number of parameter sets. Each set is used to simulate the model, producing an ensemble of time course trajectories $S_{i,t}$. These can be statistically summarised as


(3) $$\mu_{t}=\frac{1}{M}\sum_{i}S_{i,t},$$

where *M* is the number of trajectories, or visualized using built-in plotting functions to evaluate system robustness, identify sensitive parameters, or quantify variability in predicted behaviours. In addition to dynamic simulations, MxlPy supports MC-based analysis of protocol-driven simulations (e.g. experimental perturbation protocols), steady-state properties, and metabolic control coefficients. All simulations are executed via a unified interface (e.g. mc.time_course), allowing for reproducible and scalable ensemble workflows with minimal overhead.

#### 2.4.1 Case study 1: stomata opening

To illustrate the use of MxlPy’s ensemble modelling and parallel simulation capabilities, we performed a global sensitivity analysis of a classic stomatal conductance model by Kirschbaum *et al.* (1988). Stomata, microscopic pores in the leaf epidermis, respond to environmental and physiological cues, mediating gas exchange (CO_2_ uptake and O_2_ release) in plants. Kirschbaum *et al.* (1988) developed a semi-empirical mechanistic model of three ODEs, representing a not-specified biochemical signal *S* that is directly influenced by the applied light intensity, the osmotic potential (π) in the guard cells surrounding the stomata, and the leaf water potential (*w*). The stomatal conductance ($g_{s}$) is proportional to *w*. All three ODEs follow simple first-order kinetics, each of which is specified by a rate constant determining the change of the biochemical signal ($\tau_{i}$, $\tau_{d}$, for increasing and decreasing signal), the osmotic ($\tau_{\pi }$), and the water potential ($\tau_{w}$). By simulations and parameter variations, Kirschbaum *et al.* could show that their model performs reasonably well under changing light conditions and can replicate transient responses. Yet, as already stated in their discussion, the rate constants of the processes determining the stomatal conductance could vary depending on the leaf’s physiological state, hence further model evaluation should account for state-dependent kinetics.

Here, we combined MxlPy’s MC and parallel simulation facilities with sampling and analysis methods provided by the SAlib package (Herman and Usher 2017, Iwanaga et al. 2022) to conduct a time-dependent global sensitivity analysis. We aimed to find which parameter variation significantly influences the stomatal conductance in a changing light environment. Firstly, we specified parameters $\tau_{i}$, $\tau_{d}$, $\tau_{\pi }$, $\tau_{w}$ as varied input and the time-course of the stomatal conductance $g_{s}$ as analysed output. Secondly, for the input parameters, we assumed Gaussian priors with mean equal to the value given by Kirschbaum *et al.* and variation such that 99.7% of the drawn parameters are located between 80% and 120% of the original values. Thirdly, to sample the parameter space evenly, we chose Sobol sequences that minimize discrepancy (not sampled regions) in the parameter space. Lastly, we decided to use Sobol sensitivity analysis, a variance-based technique that indicates what percentage of the output variance is determined by the input variance. The Sobol method provides three different metrics: the first-order sensitivity ($S_{1}$), which indicates the influence of variance in a specific parameter on the output; the second-order sensitivity ($S_{2}$) specifies the combined effects of parameter variations; and the total-order sensitivity ($S_{T}$) combines both first- and second-order effects into one metric. Discrepancies between $S_{1}$ and $S_{T}$ are a good hint for higher-order parameter interactions determining changes in the output.

**Figure 2.**
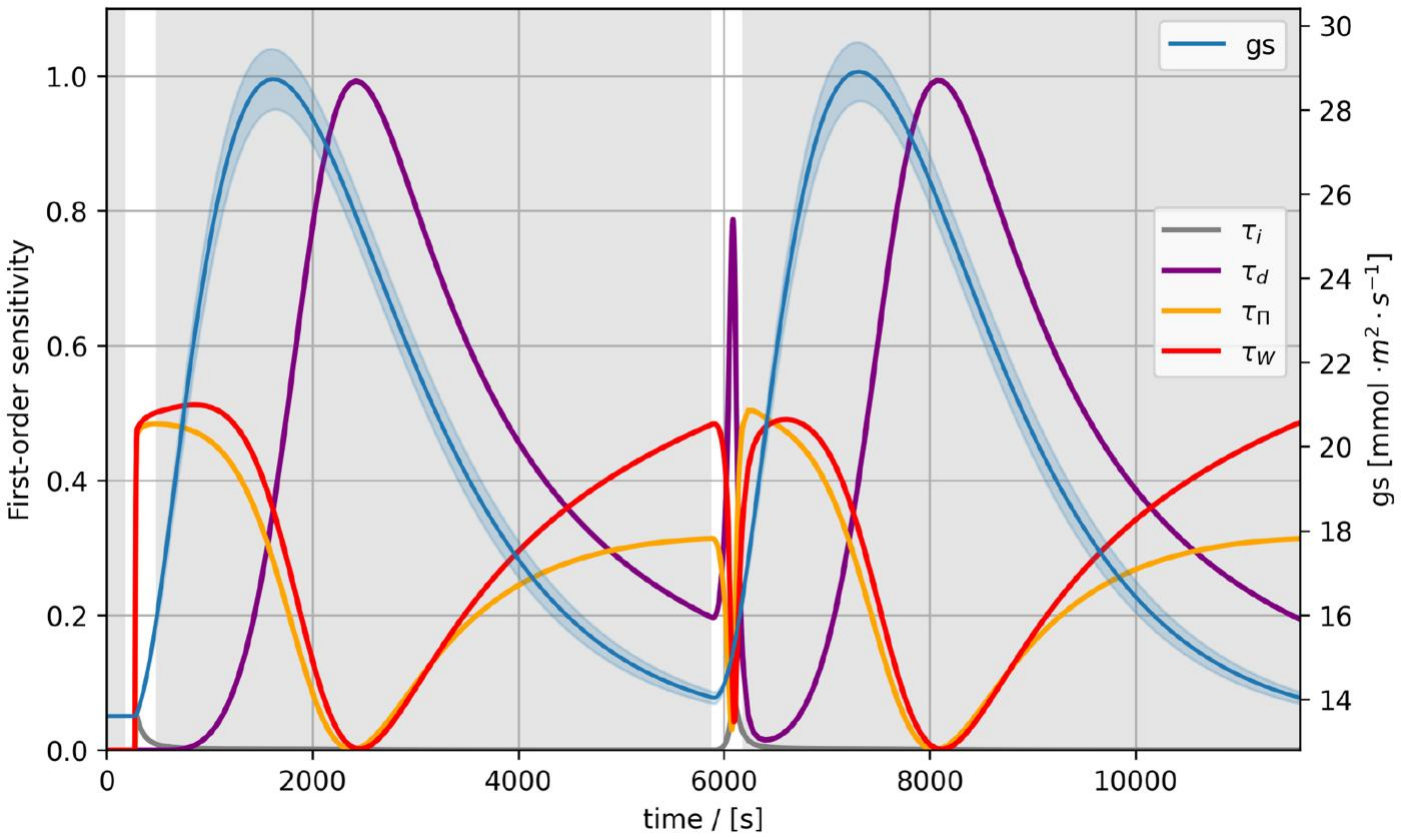
Global sensitivity analysis of the stomatal conductance by Kirschbaum *et al.* (1988). First-order sensitivities $S_{1}$ (purple, red, orange, and grey lines) and average trajectory of the stomatal conductance $g_{s}$ (blue line). Two consecutive 5 min light pulses a 500 μmol m^−2^ s^−1^ interrupted by dark phases applied to a previously dark-adapted leaf are shown. The parameters varied for the sensitivity analysis were the rate constant for the biochemical signal, the osmotic, and the water potential in the leaf ($\tau_{i}$, $\tau_{d}$, $\tau_{\pi }$, $\tau_{w}$).

We ran a simple protocol: two consecutive 5-min light pulses at 500 μmol m^−2^ s^−1^, separated by dark phases, on a dark-adapted leaf (Fig. 2). After applying a light pulse, the conductance has a delayed reaction and the chosen variation in the kinetic constants did not influence the time course drastically. Thanks to the Sobol sensitivity analysis, we could show that, from the onset of a light pulse to the dark phase, the rate constants of water and osmotic potential are responsible for the changes in the $g_{s}$ curve. Only later, the biochemical signal $\tau_{d}$ rate constant takes over and dominates the variations in $g_{s}$ shortly after its maximum. Interestingly, the regions of the highest variations in the $g_{s}$ curve are characterized by high $S_{1}$ sensitivities for all rate constants except $\tau_{i}$, which only plays a role during a light pulse (compare maximum and decline of $g_{s}$ trajectory). This case study illustrates the software’s capacity to propagate uncertainty, attribute variance over time, and surface the few rate constants that matter most for a given stimulus.

### 2.5 Multi-experiment fitting

To enable fitting ensembles, where some parameters might be shared between models, MxlPy supports multi-experiment fitting (Maiwald and Timmer 2008). To enable fitting ensembles, where some parameters might be shared between models, MxlPy supports multi-experiment fitting (Maiwald and Timmer 2008). Here, the user can choose between routines for single/multiple models, single/multiple data and single/multiple methods. This way, even complex fitting approaches, like combining steady-state and time-series data with different data sets corresponding to each, can easily be performed. All these routines are fully customizable via dependency injection. It is, thus, possible to use custom minimizers as well as overwriting the numerical integrators and residual function per routine.

### 2.6 Surrogate models integration

Besides missing parameter information, a lack of structural details (equations, network structure) significantly hinders building models. Especially for more advanced model projects with different compartments, physical, chemical or biological details are often not fully known. However, gap-free computational representations of natural processes are necessary for making precise predictions informing, e.g. medical decision-making or creation of digital twins (Kamel Boulos and Zhang 2021, Pylianidis *et al.* 2021, Attaran and Celik 2023, Purcell and Neubauer 2023). Surrogate models that utilize ML methods have been established to address information gaps by adding statistical models,


(4) $$\frac{dS}{dt}=Nv(t,S,k)+\{\begin{array}{cc} \text{NN}(t,S,k) & \left(\text{Neural}\;\text{Network}\right)\\ P(t,S,k) & \left(\text{Polynomial}\right) \end{array}$$

**Figure 3.**
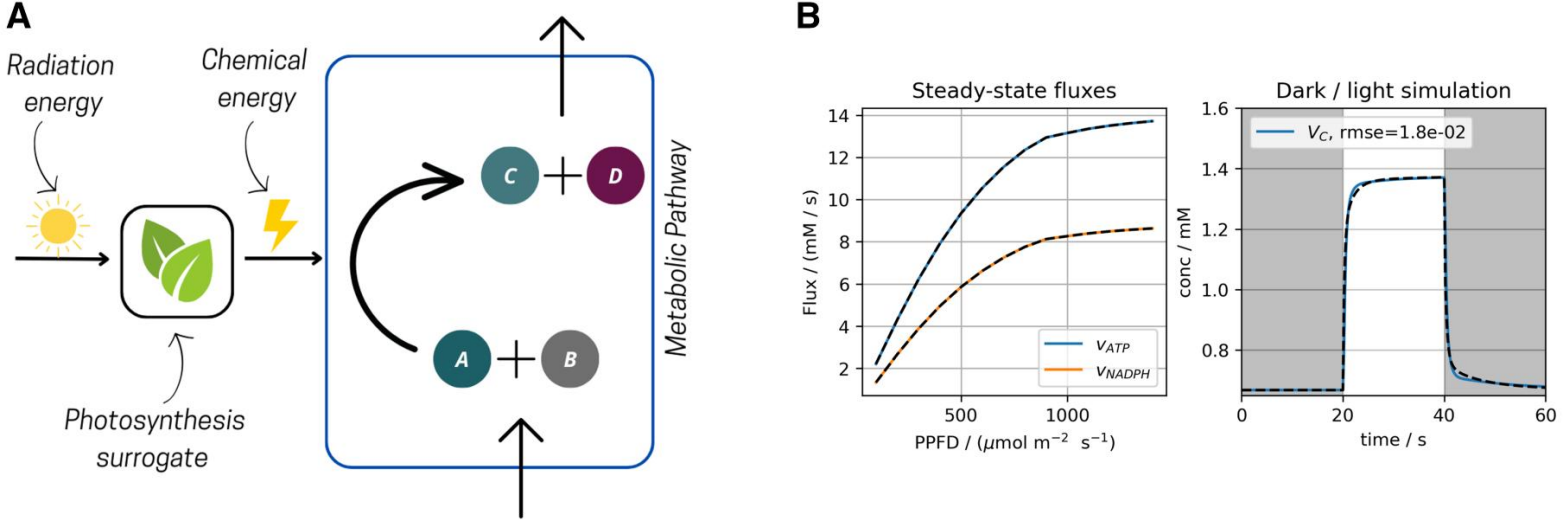
Implementing a photosynthesis surrogate. (A) Scheme of the surrogate. The details of the photosynthetic electron transport chain have been black-boxed using ML techniques. This allows for a computationally efficient way to integrate the light reactions into mechanistic models of secondary metabolism in photosynthetic organisms, maintaining complex responses to varying light environments. (B) Comparison of the original photosynthesis model and its hybrid version, where the PETC module was replaced by a surrogate. An ordinary differential equation-based model of PETC and CBB (Saadat *et al.* 2021) has been used for this analysis. Left panel: Net steady state rates of ATP and NADPH production achieved by the original and hybrid models, dependent on illumination. Right panel: Simulation of exposure to a dynamic light environment using a dark/light/dark protocol. Shown is the RuBisCO carboxylation rate $V_{C}$ as predicted by the ODE model (blue line) in comparison to the hybrid model (dashed black line).

Moreover, surrogate models can replace mechanistic model parts in intensive simulations due to their lower computational cost after training. MxlPy allows users to train surrogates and integrate them into models. These can range from simple polynomials and cubic splines to deep neural networks or custom user-defined functions. For NNs, we support all popular libraries as backends, namely PyTorch (Paszke *et al.* 2017), TensorFlow (Abadi *et al.* 2015), Keras (Chollet *et al.* 2015), and Equinox (Kidger and Garcia 2021). Using training data, for example, concentrations of substrates and metabolic fluxes, a multi-layer perceptron is trained for a specified number of epochs. Train types (full batch, mini-batch) and hyperparameters are customizable. Once a surrogate is trained, MxlPy allows the integration of surrogates in an existing model with the add_surrogate method of a model object. Surrogates could, for example, be used in photosynthetic organism models simulating anabolic pathways, e.g. chlorophyll synthesis. Surrogates trained on detailed, pigment dependent models of light reactions (e.g. Pfennig *et al.* 2024) would allow for computationally cheap estimations of reactant productions, fuelling the anabolic model (see Fig. 3). This would particularly improve performance when the models differ in time resolution. Beyond computational efficiency, such surrogate integration enables biotechnological applications, such as the rational design of strains optimized for metabolite production under specific light conditions. By tailoring models based on the availability of energy carriers like ATP and NADPH, researchers can better predict and engineer productivity in photosynthetic systems.

#### 2.6.1 Case study 2: linking photosynthetic scales

Photosynthesis is a vital biochemical process that sustains life on Earth. Plants, algae, and cyanobacteria capture solar energy and powered by the photosynthetic electron transport chain (PETC) use it to fix carbon dioxide via the Calvin-Benson-Bassham cycle (CBB) to other organic compounds that are the building blocks of biomass. To demonstrate the procedure of surrogate integration, we developed a surrogate of the PETC part in a model of photosynthesis, that included both electron transport and downstream carbon assimilation (Saadat *et al.* 2021). In this example, a surrogate approximates the steady state production of ATP and NADPH in the PETC. This approximation serves as input for the CBB cycle module. As Fig. 3 shows, the hybrid model (including the surrogate instead of the full dynamic description of the PETC) reproduced the behaviour of the original model. Moreover, in terms of computational efficiency, the hybrid model could speed up the runtime (machine-dependent) of the full model by a factor of 2.6 (measured as average over 1000 simulations).

### 2.7 Neural posterior prediction

NPE is a technique that, given a model structure and parameter priors, learns to predict the parameter posterior distribution. The frequent intractability of the data likelihood function in model studies, which is needed for classical Bayesian approaches, motivated researchers to develop computational advanced simulation-based inference techniques (SBI) consisting of Approximate Bayesian Computation and neural density estimation (Sunnåker *et al.* 2013, Papamakarios and Murray 2018, Greenberg *et al.* 2019, Papamakarios *et al.* 2019, Cranmer *et al.* 2020, Hermans *et al.* 2020). NPE circumvents the regular Bayesian workflow of collecting priors, deriving a likelihood function, and calculating the posterior via the Bayesian formula, using a NN instead. This NN directly predicts the posterior distributions of model parameters given the data. It is trained on a model-informed synthetic data set combined with parameter priors.


(5) $$\underset{\text{Prior}}{\underset{︸}{p(k)}}\times \underset{\text{Likelihood}}{\underset{︸}{p(S|k)}}\overset{\text{Neural}\;\text{Network}\;}{\to }\underset{\text{Posterior}}{\underset{︸}{p(k|S)}}$$

MxlPy provides an easy-to-use npe module that allows users to specify simulation-based inference, particularly NPE workflows. For this, the users must first specify a suitable prior distribution for the model parameters. The prior distribution can be derived from database information using MxlPy’s parameterisation functionalities or domain knowledge. A synthetic data set is created using the prior distributions by leveraging MxlPy’s scan and distributions modules, facilitating the sampling of parameters and simulations over a range of parameter values. Simulated data, such as time courses, steady states, control coefficients, or summary statistics, is then used to train a NN that predicts a parameter set given a synthetic data sample. For this, the user can choose one of the npe module’s training functions, allowing access to PyTorch. The trained NN can be used as an approximation for a posterior distribution function, describing the parameter probability for a given data set via density estimation. The default NN in the npe module is the same as described for the surrogate module (see above), but can be changed by the users to use state-of-the-art NN architectures presented in the SBI literature.

## 3 Discussion and future prospects

To our knowledge, MxlPy is the first Python framework that seamlessly integrates ML into systems biology modelling, offering users new possibilities for mechanistic model construction and analysis. While it shares aspects with other computational tools—such as CADET for chromatography and bioprocess modelling (Leweke and von Lieres 2018), classical toolboxes like Copasi, Tellurium, BioNetGen, or VCell (Blinov *et al.* 2004, Hoops *et al.* 2006, Choi *et al.* 2018), and recent Python projects like MEMMAL (Erdem and Birtwistle 2023), MxlPy distinguishes itself through its Pythonic, easy-to-use architecture specifically designed to integrate ML and ensemble modelling into mechanistic workflows. No domain language is established to describe and formalize computational models, allowing users to employ their knowledge of Python. Additionally, MxlPy shares common ground with the HybridML project (Merkelbach *et al.* 2022). Both tools allow the integration of NNs into the model process. However, MxlPy emphasizes but is not constrained to metabolic models. MxlPy allows, for instance, metabolic control analysis in ensemble modelling to identify critical steps in industrial or medically relevant metabolic pathways with uncertain parameters. In the Julia ecosystem, SciML/Catalyst (Loman *et al.* 2023) serves an analogous purpose for symbolic reaction networks. While scientific ML broadly focuses on embedding physical or biological constraints into neural architectures, MxlPy was created to serve the specific and practical needs of life science modellers—offering a transparent, modular framework that supports end-to-end workflows for kinetic model construction, calibration, hybridization with ML components, and reproducible analysis within the context of complex experimental collaborations.

## Supplementary Material

vbaf294_Supplementary_Data
