# Supplementary material for: MxlPy—Python package for mechanistic learning and hybrid modelling in life science
Source: Bioinform Adv. 2025 Nov 18;5(1):vbaf294. doi: 10.1093/bioadv/vbaf294 (PMC12668773; doi:10.1093/bioadv/vbaf294)
Supplement: vbaf294_Supplementary_Data [file vbaf294_supplementary_data.pdf]

Supplementary information - MxLpy - python package for mechanistic learning in biology and medicine

Marvin van Aalst Tim Nies Tobias Pfennig Anna Matuszyńska

1 Software Comparison

The following table compares various software outlining the need for frameworks that allow easy integration of machine learning/ neural networks into mechanistic models.

We assumed a feature would be available if a function in the software allowed specific analyses (either as direct implementation or wrapper for third-party software). However, if just examples were given in the documentation of using third-party software directly, we defined a feature not available in the software itself. Stability analysis refers to linear stability analysis of complex systems. N/A indicates that we could not find any indicator that the feature is implemented.

Although care was taken to find all relevant information in the documentation of each software, the reader is referred to each software's website for detailed descriptions of its functionalities.

Table S1: Comparison of modeling software platforms.

**Software**	**MxlPy**	**HybridML**	**Catalyst.jl**	**Copasi**	**VCell**	**Tellurium**	**CADET-suite**
				**General**			
Research Area	Life science	Chemical engineering, Life science	(Bio)-chemistry	Life science	Life science	(Bio)-chemistry	Bio-/Chemical engineering (Chromatography)
First publication year	2025	2022	2023	2006	1997	2016	2018
Maintenance (last update)	Apr. 2025	Jan. 2023	Apr. 2025	Jul. 2024	Jul. 2024	Jan. 2024	Apr. 2025
Open Source	Yes	Yes	Yes	Yes	Yes	Yes	Yes
Language	Python	Python	Julia	C++ (parts in C, Perl, bindings available)	Java (parts in Python, Perl)	Python (libRoadRunner C++)	C++ (bindings available)
Model import	SBML	Json	SBML, .net (BioNetGen), .jl Julia Files, .jls Julia Serialization	SBML, Copasi file .cps, Gepasi, SED-ML files, COMBINE Archives	SBML, VFRAP, VCML (Virtual Cell Markup Language), BNGL (BioNetGen Language)	SBML, CELLML, SED-ML, COMBINE, SBOL, Antimony	HDF5, XML
Model export	SBML	Json	.jl Julia Files, .jls Julia Serialization	SBML, C source files, XPPaut, Berkley Madonna files	SBML, SEDML, VCML, Matlab files, Smoldyn files	SBML, COMBINE, Antimony, CellML, Matlab	HDF5, XML
Text summaries	LaTeX	N/A	LaTeX	Various formats	Various formats	N/A	N/A
Link	`https://github.com/Computational-Biology-Aachen/MxlPy`	`https://github.com/ARC-COMBINE/HybridML`	`https://github.com/SciML/Catalyst.jl?tab=License-1-ov-file`	`https://copasi.org/`	`https://vcell.org/`	`https://tellurium.analogmachine.org/`	`https://cadet.github.io/master/index.html`
				**Mechanistic models**			
ODE solver	various Solver (Scipy, CVODE)	Casadi	Various solvers (DifferentialEquation.jl)	LSODA, LSODAR	Various solvers (IDA, CVODE, etc.)	various Solvers (libRoadRunner, Scipy)	IDAS
PDE solver / Grid methods	N/A	N/A	Spatial ODEs module (Grid methods)	N/A	Various solvers (based on finite volume or Smoldyn - Smoluchowski Dynamics)	N/A	Grid methods
Sensitivity Analysis methods	Yes	N/A	Yes	Yes	Yes	Yes	Yes
Monte-Carlo methods	Yes	N/A	Yes	Yes	Yes	Yes	Yes
Plotting methods (not third parties)	Yes	N/A	N/A	Yes	Yes	Yes	Yes
Model parametrisation (fitting)	Yes	N/A	Yes	Yes	Yes	Yes	Yes
Stability Analysis	Yes	N/A	Yes	Yes	N/A	Yes	N/A
				**Mechanistic learning**			
ML/DL Framework	PyTorch	Tensorflow	Surrogates.jl, SciMLSensitivity.jl, and DiffEqFlux.jl.	N/A	N/A	N/A	N/A
Surrogates	Neural Networks, Polynomial	Neural Networks	data-driven models	N/A	N/A	N/A	N/A
Simulation-based inference	Neural posterior estimation	N/A	N/A	N/A	N/A	N/A	N/A
